# Correction: Biscoumarin-containing acenes as stable organic semiconductors for photocatalytic oxygen reduction to hydrogen peroxide

**DOI:** 10.1039/d5ta90079d

**Published:** 2025-04-08

**Authors:** Marek K. Węcławski, Marie Jakešová, Martyna Charyton, Nicola Demitri, Beata Koszarna, Kerstin Oppelt, Serdar Sariciftci, Daniel T. Gryko, Eric Daniel Głowacki

**Affiliations:** a Institute of Organic Chemistry, Polish Academy of Sciences Kasprzaka 44/52 Warsaw Poland dtgryko@icho.edu.pl; b Linz Institute for Organic Solar Cells (LIOS), Physical Chemistry, Johannes Kepler University Altenbergerstrasse 69 A-4040 Linz Austria; c Laboratory of Organic Electronics, ITN Campus Norrköping, Linköpings Universitet Bredgatan 33 S-602 21 Norrköping Sweden eric.glowacki@liu.se; d Elettra – Sincrotrone Trieste SS14, Km 163.5 34149 Basovizza TS Italy; e Institute of Inorganic Chemistry, Johannes Kepler University Altenbergerstrasse 69 A-4040 Linz Austria

## Abstract

Correction for ‘Biscoumarin-containing acenes as stable organic semiconductors for photocatalytic oxygen reduction to hydrogen peroxide’ by Marek K. Węcławski *et al.*, *J. Mater. Chem. A*, 2017, **5**, 20780–20788, https://doi.org/10.1039/C7TA05882A.

The authors regret that by oversight they twice added the same UV-vis absorption spectrum of the evaporated film of compound **9** in Fig. 2, and the correct UV-vis spectrum of the evaporated film of compound **8** was missing. The corrected Fig. 2 should appear as shown below:
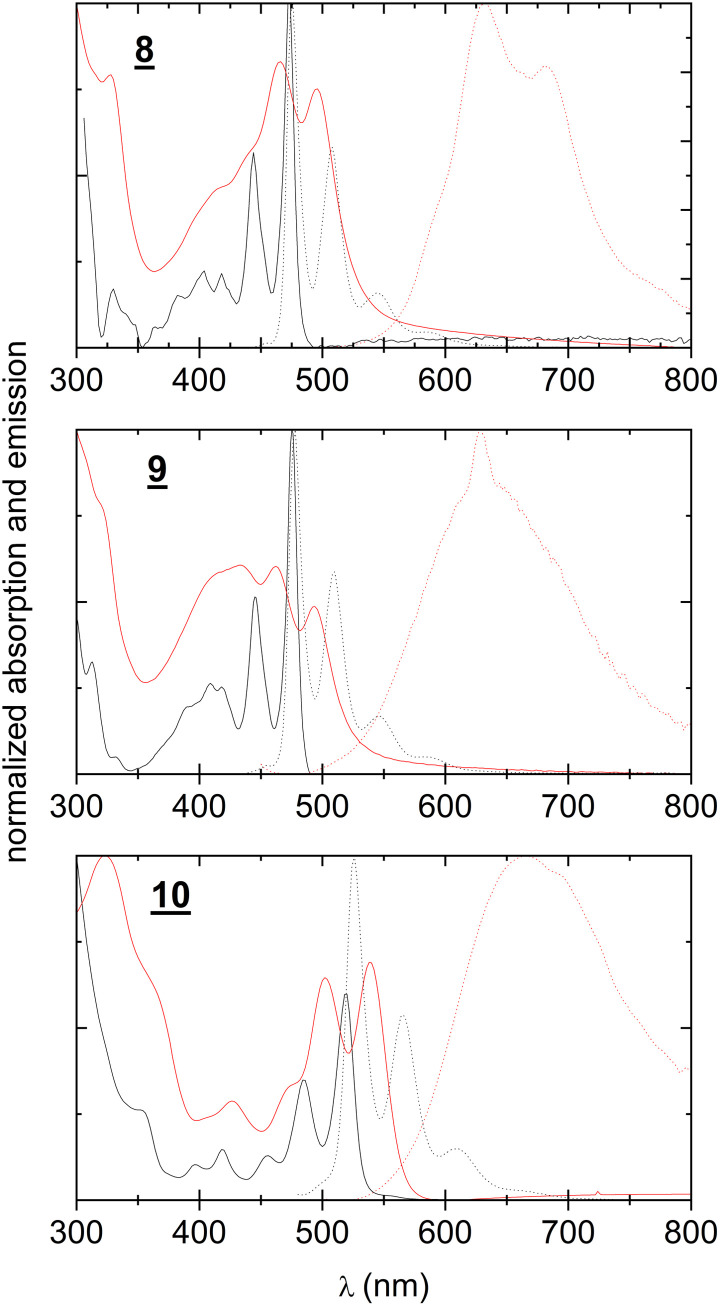


**Fig. 2** UV-visible absorption and photoluminescence spectra of **8–10**. Black continuous line: absorption of solutions, 0.1 mM in toluene. Black dotted line: photoluminescence of the same solutions. Red continuous line: absorption of evaporated thin films. Red dotted line: photoluminescence of the same films.

The authors apologize for this mistake, and maintain that no critical conclusions or concepts of the paper were significantly affected by the missing spectrum of the evaporated compound **8**.

The Royal Society of Chemistry apologises for these errors and any consequent inconvenience to authors and readers.

